# Bioconversion of Keratin Wastes Using Keratinolytic Microorganisms to Generate Value-Added Products

**DOI:** 10.1155/2022/2048031

**Published:** 2022-03-22

**Authors:** Muhammed Seid Anbesaw

**Affiliations:** Wollo University, School of Bio-Science and Technology, Department of Biotechnology, Dessie, Ethiopia

## Abstract

The management of keratinous wastes generated from different industries is becoming a major concern across the world. In each year, more than a billion tons of keratin waste is released into the environment. Despite some trials that have been performed and utilize this waste into valuable products, still a huge amount of keratin waste from different sources is a less explored biomaterial for making valuable products. This indicates that the huge amount of keratin waste is neither disposed properly nor converted into usable products rather thrown away to the environment that causes environmental pollution. Due to the introduction of this waste associated with different pathogenic organisms into soil and water bodies, human beings and other small and large animals are affected by different diseases. Therefore, there is a need for modern and ecofriendly approaches to dispose and convert this waste into usable products. Hence, the objective of this review is to give a concise overview regarding the degradation of keratin waste by biological approaches using keratinase producing microorganisms. The review also focuses on the practical use of keratinases and the economical importance of bioconverted products of keratinous wastes for different applications. Various researches have been studied about the source, disposal mechanisms, techniques of hydrolysis, potential use, and physical and chemical properties of keratin wastes. However, there is negligible information with regard to the use of keratin wastes as media supplements for the growth of keratinolytic microorganisms and silver retrieval from photographic and used X-ray films. Hence, this review differs from other similar reviews in the literature in that it discusses these neglected concerns.

## 1. Introduction

**Figure 1:**
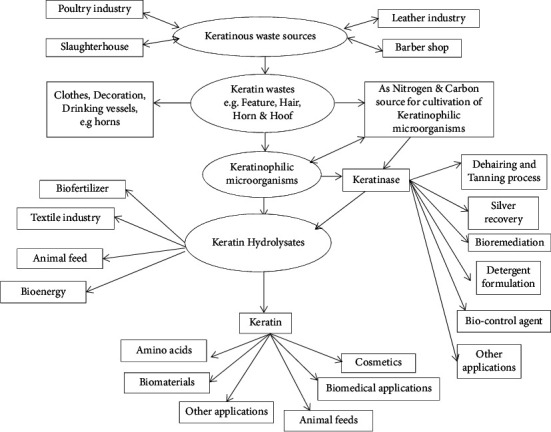
Summarized schematic illustration of integration between sources of keratin wastes, bioconversion of keratin wastes, generation of value-added products, and their applications (source: author).

Human civilization with numerous activities results in accumulation of a huge amount of solid wastes in the environment. With the expansion of the human population, disposal and management of solid waste is becoming one of the major alarms faced by the human race. Keratin wastes are one of the solid wastes released to the environment, and only a part of the discarded materials are recycled or subjected to compost; most are landfilled or incinerated [1]. Each year, billions of kilograms of keratin wastes are produced from different sectors (Figure 1). These include the poultry industry, leather industry, meat industy, and barbershops. These organizations release huge keratinous wastes to the surrounding and create a serious solid waste management problem in many countries and cause serious environmental pollution [2].

Despite different disposal mechanisms being developed to minimize keratin wastes, such as dumping in appropriate places, buried, used for landfilling, or incinerated, annually a very large amount of keratin waste is produced worldwide. Keratin wastes in the form of feathers, hairs, horns, nails, and hooves are generated mainly from animal body parts [3] and as a waste byproduct of industrial processes mostly from the slaughterhouses, poultry farms, and leather industries [4]. The accumulation of these wastes in the environment is considered environmental contaminants and upturns the threats of environmental risks [4, 5].

After cellulose and chitin, keratin is the third most abundant polymer in the environment [6]. Many animals' hairs, feathers, hooves, nails, wools, and horns are primarily composed of keratin, a fibrous structural protein that belongs to a broad family of fibrous structural proteins [4, 7]. Keratin proteins are biochemically inert biomaterials and are difficult to solubilize under mild conditions. This is due to the existence of a large number of disulfide bonds between cysteine amino acids in keratin materials [4, 8, 9]. Therefore, keratin is difficult to degrade completely into small components especially by common proteolytic enzymes (trypsin, pepsin, papain, and bromelain), which are mainly obtained from plant sources. Almost all the reports [10–14] indicate that complete degradation of keratin wastes is accomplished by microorganisms and their enzymes rather than plants and their enzymes.

**Figure 2:**
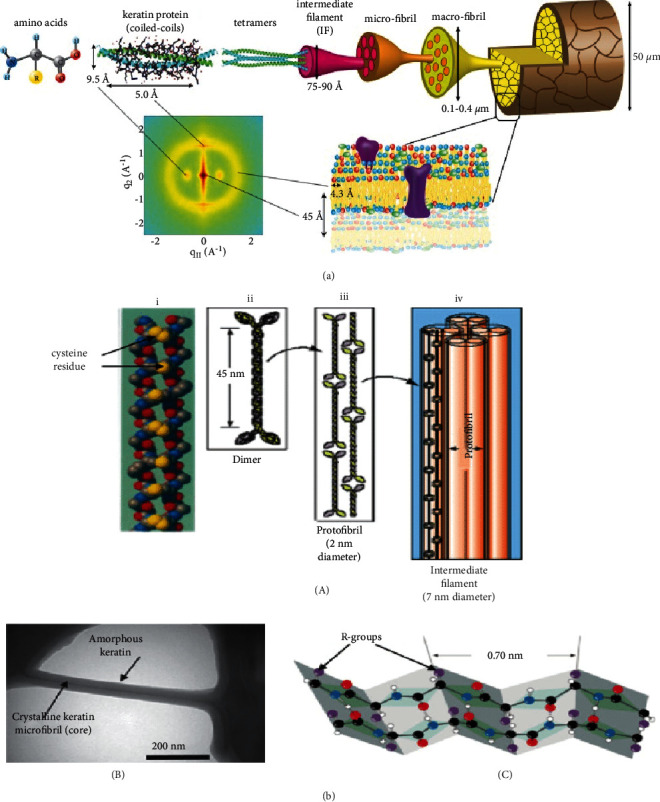
Structure of keratin [16]: (a) hierarchy of *α*-keratin showing the assembly from two polypeptide chains (i) to a fibrous structure (iv), (b) TEM micrograph of *α*-keratin from a sheep horn displaying the composite structure of a crystalline keratin core within an amorphous keratin matrix, and (c) *β*-keratin with a pleated sheet shape that consists of anti-parallel chains with R-groups that extend between sheets [17].

Based on cysteine contents, keratin can be divided into soft and hard keratins. Keratins with cysteine content of less than 10% are termed soft keratins, whereas those with cysteine content between 10% and 14% are termed hard keratins [15]. Hard keratin typically exists in nails, hair (Figure 2(a)), claws, hooves, and wool, whereas soft keratin is found in the soft tissues including the epidermis of the skin. *α*-keratin and *β*-keratin (Figures 2(a)–2(c)) are the two major subdivisions of keratin materials based on their secondary structure or the arrangement of the amino acid chain [18, 19]. If the secondary structure of keratin contains *β*-pleated, it can be termed *β*-keratins (Figures 2(b) and 2(c)), while if it contains *α*-keratins, it is considered *α*-keratins (Figures 2(a) and 2(b)). As Fang et al. [20] reported that the composition of *α* and *β* keratins are dissimilar among many organs. For instance, wool generally contains *α*-keratin, while feathers contain both *α*- and *β*-keratins [21]. *α*-keratins are slightly basic, and they form a right-handed helix, while *β*-keratins are slightly acidic, and they form a left-handed helix [22–24].

The amount of cysteine content present in keratin materials results in a variation of the hardness of these materials. Therefore, keratins can be classified into hard and soft keratins based on their chemical composition, the total amino acid residues, and mainly the amount of cystine. Keratin containing up to 2% of cysteine in its composition is termed as soft, and keratin containing cystine up to 22% is considered as hard keratin [25]. Horn, nails, hair, feather, claw, and wool are examples of hard keratin, and skin and hair core are examples of soft keratins. Hair and wool (10–17% cysteine content) are typically hard keratins, as are feathers (4–8% cysteine content), and skin is soft keratin (2% cysteine content) [26, 27]. This implies that hard keratin contains high sulfur content, while soft keratin contains a small amount of sulfur [28].

## 2. Impact of Keratin Wastes on Environmental Pollution and Human Health

Keratin wastes can be generated in huge quantities from the poultry industry, tannery industry, wool industry, textile industry, abattoir house, and barber and hairstylist shops. These wastes have the potential to pose a possible danger to biotic and abiotic components of the environment. This includes pollution of soil, air, and water as well as animal health problems [29, 30].

Conventional disposal and management of keratin wastes such as dumping, burying, landfilling, and incinerating are applicable for a century to reduce the excess amount of these wastes. However, all these methods cannot safeguard the environment from pollution rather they affect public health and increase the risks of environmental hazards as well as greenhouse gases concentration [2]. Therefore, disposal of a huge volume of unusable keratin materials released mostly from the tannery industry, poultry industry, and abattoir house is considered environmental pollutants and causes a major solid waste problem in many countries. Keratin wastes including feather and other keratin-containing wastes are associated with the development of odors and infectious agents in the air, water, and soil and cause environmental problems, as they tend to accumulate. One of the inappropriate disposal mechanisms of poultry feathers causes various human health problems including chlorosis and fowl cholera due to the presence of pathogenic microflora [4, 31, 32].

In general, wastewater containing keratin wastes causes environmental pollution as well as negatively affects the resident's life living in proximate areas. The waste also causes the problems of acidification of soils, eutrophication, and decreased species diversity [4]. Therefore, well-organized and instant action on keratin wastes management should be planned by concerned bodies.

## 3. Management Options of Keratin Wastes

**Figure 3:**
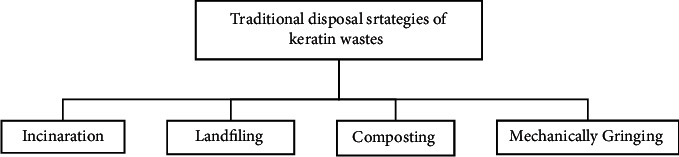
Management options of keratin wastes.

Despite their drawback, there are a number of conventional keratin wastes management strategies. Incineration, landfilling, composting, and mechanically grinding are the major ones [12] (Figure 3). Disposing of keratin wastes with these approaches is not advisable due to excess releasing of harmful gases to the environment and causes different problems for aquatic and terrestrial animals [33]. Considering the adverse effect of the above all keratin wastes disposal approaches, scientists try to investigate other environmental friendly approaches to manage these wastes using microorganism [12] because the negative impact of conventional management of keratin wastes on the environment and human health can be reduced to a great extent by using microorganisms that have the capability to compose keratin materials [4]. Thus, from an environmental and economic point of view, the management of keratin wastes using microorganisms (keratinophilic microorganisms) is the best alternative option to manage keratin wastes properly and use these wastes as a raw material to produce useful products for different applications [12].

## 4. Approaches to Convert Keratin Wastes into Keratin Hydrolysate

**Figure 4:**
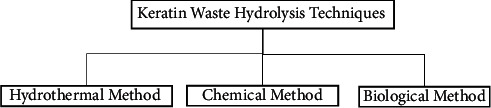
Approaches to convert keratin wastes into keratin hydrolysates.

Several attempts have been made to degrade keratinous wastes to ensure the appropriate utilization of keratin protein for different industrial purposes. Hydrothermal, chemical, enzymatic, or biological treatments (Figure 4) are the well-known conventional techniques that have been developed to hydrolyze keratin wastes [13].

### 4.1. Hydrothermal Treatment Approaches

The main drawback of hydrolysis of keratin wastes by the hydrothermal process is The main disadvantage of hydrothermal keratin waste hydrolysis is that temperature-sensitive peptides containing methionine, lysine, and tryptophan (essential amino acids) are partially or completely destroyed, resulting in the creation of lanthionine, lysinoalanine, and other compounds (nonnutritive amino acids). Not only this, but also the process is also frequently carried out in the presence of strong acids and bases at high temperature (80–150°C) and/or high steam pressure (10–15 psi), which is highly expensive [12, 34]. Therefore, this process is energy-intensive and also creates an additional pollution burden [13, 35].

### 4.2. Chemical Treatment Approaches

Chemicals are one of the traditional methods for hydrolysis of keratin wastes (acid, base, and catalyst). For processing, this method requires more time, concentrated chemicals, and energy, as well as unaffordable industrial instruments. Since this process needs extremely hostile reaction conditions (high pressure and temperature), it may affect the environment adversely [36]. The chemical hydrolysis process causes respiratory disorders, cardiovascular diseases, and cancer, among other diseases, by increasing the emission of various gases such as CO_2_ and SO_2_ into the environment [37]. The product of chemical hydrolysis of keratin wastes has little nutritional value because it contains limited levels of essential amino acids [38]. Hence, an immediate action is needed that is a highly efficient, cheap, and environmental friendly approach to processing keratinous wastes.

### 4.3. Enzymatic or Biological Treatments

Biotechnological approaches using microorganisms and their keratinolytic enzymes have been used to upgrade the commercial value of keratinous wastes for different applications [12, 39]. Thus, advances in microbial enzyme technology offer substantial opportunities for the development of green evolutions. Bioconversion of keratin wastes into usable or value-added products (Figure 1) by low-energy consuming technologies is one aspect of the promising benefit of microbial enzyme technology [12, 40].

Biodegradation of keratin wastes by keratinophilic microorganisms or their enzymes (keratinases) overcomes the drawbacks observed by chemical and thermal treatments [4]. This biotechnological and eco-friendly alternative for hydrolysis and recycling of keratin wastes is not only improves the commercial value of keratin wastes but also offers mild conditions for the production of valuable products. Biological keratin waste degradation is a modern approach that is more effective than chemical and hydrothermal keratin waste degradation in terms of cost and environmental concerns. The product (keratin hydrolysate) obtained by this method is toxin-free and can be used in commercial applications [4]. Therefore, bioconversion/biological degradation of keratin wastes is a novel technique for degrading and using keratin wastes as a valuable biomaterial in terms of cost-effective and environmentally friendly processing, as well as producing commercially useful byproducts that can be used in a wide range of applications. As [6, 41] investigated, the mechanism of biodegradation of keratin materials needs the synergistic action of keratinophilic microorganisms and their extracellular keratinase enzyme. This implies that neither keratinophilic cells nor keratinase alone can degrade native keratin materials completely. Hence, the presence of both living cells and the enzyme keratinase is important for the complete degradation of keratin materials [42–44].

**Figure 5:**
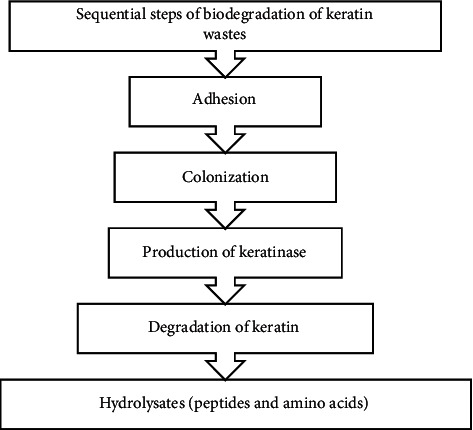
Enzymatic or biological treatments of keratinous materials [43].

**Figure 6:**
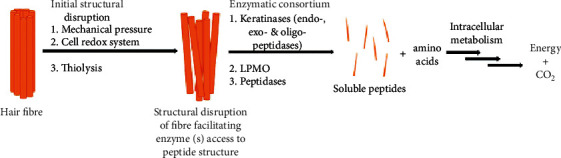
Possible mechanisms for microbial degradation of keratin (LPMO, lytic polysaccharide monooxygenase [6].

Reports proved that keratinnolytic microorganisms and their enzymes can degrade both soft and hard keratins [25, 45]. But, to date, the precise mechanism and mode of action of keratinophilic cells and their enzymes on intact keratin biodegradation are still not completely well known. However, Peng et al. [43] recognized that bioconversion of keratin material follows mainly four major sequential steps. These are adhesion, colonization, production of keratinase, and breakdown of the keratin substrate (Figure 5). In the first step of the keratin biodegradation process, the microbial biomass adheres and colonizes to the surface of keratin material, biomembrane potential theory [46]. The interaction of keratinophilic cells with keratin material primarily aids in the degradation of lipid components on the material's surface as well as the breaking of disulfide bonds. This means that in the biological keratin degradation process, microorganisms first consume lipids (nonkeratin components) before starting to degrade a protein component or keratin [47]. The cracking of disulfide bonds results in denaturation or loss of native keratin structure, which is an essential step for the biodegradation process. This is perhaps due to the unceasing supply of reducing agents secreted by keratinophilic cells to break disulfide bonds [48, 49]. Therefore, it is proved that the reduction of disulfide bonds is mandatory for the effective hydrolysis of intact keratin materials. After denaturation of native keratin structure, keratinase, produced by keratinophilic microorganisms, cleaves the peptide bonds and releases different amino acids and peptides [43, 50, 51] (Figure 6).

To completely degrade keratin and convert it to small entities (amino acids and peptides) and energy, three major phases must be completed: denaturation, decomposition, and transamination [52] (Figure 6). Due to the complexity of keratin, these processes (complete keratin biodegradation) are summarized into two key actions: sulfitolysis (breakdown of disulfide bonds) and proteolysis (proteolytic attack) by keratinolytic proteases (keratinases) [6, 53]. Sulfitolysis is the main process of keratinolysis [54]. In this particular process, microbes releases sulfide, which is responsible for the breakdown of disulfide bonds that exist in keratin materials (thiolysis theory), explained by Kunert et al. [55]. Due to their capability to release extracellular keratinolytic enzymes into the culture medium, bacteria and fungi have the ability to degrade keratinous substrates proficiently in the proteolysis process [6, 43, 56].

Keratinases (E.C. 3.4.21) are extracellular enzymes used for the biodegradation of keratinous materials [10, 37]. Keratinases can be produced from microorganisms of the three domains of life: eukarya, bacteria, and archaea. This confirms that keratinophilic microorganisms are very widespread in the microbial world. However, microorganisms with keratin decomposition ability are mainly bacteria [57], actinomycetes [58], and fungi [59]. These organisms commonly exist in a place where keratinous materials are deposited such as soils and aquatic environments [25, 60] including decomposing feathers [51, 61, 62], slaughterhouse polluted water [63], alkaline soda lake [62, 64], and soil of decayed keratin waste heaps. This means that the widespread existence in the nature of bacteria that grow swiftly and, in some cases, preferentially on keratinous substrates has supported the widely held belief that microbes that produce keratinolytic enzymes may destroy keratin.

Most of the keratinolytic enzyme-producing bacteria are grouped under Gram-positive bacteria, predominantly the genus *Bacillus* [65]. Bacterial species that belong to this genus and have the potential to produce keratinase includes *Bacillus subtilis*, *Bacillus pumilus*, and *Bacillus licheniformis* [66–70]. Among the well-known species, *B. licheniformis* is the most potent keratin-degrading bacterium in the genus [71]. *Streptomyces* species such as *Streptomyces griseoaurantiacus* E5 and *Streptanthus albidus* E4 [61]; *Streptomyces pactum*, *Scaphirhynchus albus*, *Streptomyces thermoviolaceus*, *and S. albus* AZA [72]; and *Streptomyces albidoflavus* [73] are also known to produce keratinolytic enzymes. Gram-negative keratinolytic bacteria, such as *Vibrio* sp. strain kr2 [74] and *Vibrio* sp. strain R11 [62], have been identified from decomposing feathers [65, 74]. Riffel and Brandelli [65] found that the majority of keratinophilic bacteria isolated from feathers decomposing area were Gram-negative.

*Rhizomucor* sp. *and Aspergillus* sp. [59], *T. loubieri* RC-S6 [75], *T. mentagrophytes*, *T. rubrum*, *T. gallinae*, *M. canis*, and *M. gypseum* [76] are some examples of the fungi species that can produce keratinolytic protease enzymes.

Actinomycetes, mainly *Streptomyces* genus, have also been described as keratinase producers. This group of bacteria, isolated from different soil sites, are accompanied the keratinolysis of various keratin materials such as wool, feathers, and hair. Actinomycetes from the *Streptomyces* group, namely *Streptomyces* sp. A11 [77], Streptomyces *thermoviolaceus* SD8 [78], *Streptomyces fradiae* [58], *Streptomyces graminofaciens* [79], and Streptomyces *pactum* [80] and the *Thermoactinomyces* group, namely, *Thermoactinomyces* sp. [81] and *Thermoactinomyces candidus* [82] are generally described as keratin degraders with the capability to act on a wide variety of keratinous materials, including wool, hair, and feathers. The thermophilic *Microbispora aerata* IMBAS-11A [83] and the mesophilic *Streptomyces flavis* 2BG [81] are the two exemplary actinomycetes having the capability to degrade keratin waste, isolated from Antarctic soil. They were cultivated for 1 day at 55°C (*Microbispora*) or at 28°C (*Streptomyces*) in mineral salt (MS) medium with milled wool waste as a sole source of nitrogen and carbon. Maximum keratinolytic activity was detected on days 5 and 8 of cultivation for strain 11A and 2BG, respectively.

Keratinase production is also associated with *Hyperthermophilic archaea* (e.g., *Aeropyrum*, *Thermococcus*, and *Pyrococcus*) and displayed their maximal activities at even higher temperature (90–110°C) [84, 85].

### 4.4. New Prospects on Keratinolytic Microorganisms and Their Keratinase

Even though there are safety issues with regard to modified microbes and their genes are acceptable in different industrial applications, researchers devoted their time to developing improved keratinolytic microorganisms and their keratinolytic enzymes to enhance the catalytic activity and thermal stability of the enzyme. Whole-cell mutagenesis and protein engineering are the two major approaches that scientists follow to achieve the intended objectives. Representative studies including [86, 87] were applied on mutagenesis to improve the target keratinolytic microorganisms and their enzymes. As de Paiva et al. 2018 [86] reported, the whole-cell mutagenesis study using ethyl methanesulfonate (EMS) as a mutagen reveals that the mutant strain of *Bacillus subtilis* LFB-FIOCRUZ 1266 was showed higher in degradation of the feather as compared to the wild-type strain. Protein engineering in particular rational protein design also plays a pivotal role in improving targeted microbes and their enzymes [88]. With the help of 3D keratinase structure and its amino acid sequence, it is possible to improve the activity and thermal stability of a keratinase with this method. This strategy has been fruitfully applied to the keratinolytic enzyme of *Bacillus licheniformis* BBE11 and the mutant keratinolytic enzyme showed 5.6-fold increase in catalytic efficiency [89]. Other approaches such as PCR-based techniques and direct evolution were also applied to create more potent keratinolytic enzymes [90].

Currently, genetic engineering is becoming one alternative way of producing industrially important keratinolytic enzymes. The technology has been used to clone keratinase genes of bacteria, fungi, and archaea and overexpressed them in host cells like *E. coli* and other host cells [91].

## 5. Commercial Value of Keratin Wastes/Hydrolysates

Human civilizations have a long history of using keratinous materials for the production of daily life utensils and curios, such as the use of mammalian and reptile skin as leather covers and clothing, feathers as different bedding materials and clothing, and horn sheaths as drinking vessels among many others [37]. Even though keratinous materials have a diversified commercial value for different applications (Figure 1), we are throwing away it as a waste byproduct yet. But now, fruitful tries have been made to renovate keratinous wastes such as animal hair, horns, poultry feathers, and hooves into value-added products by keratinophilic microorganisms.

### 5.1. A Source of Nitrogen and Carbon for Cultivation of Microorganisms

With the emergence of microbial biotechnology, there is a greater demand for high-quality, low-cost microbial growth media. The utilization of keratinous wastes from various industries, such as poultry industries, barbershops, slaughterhouses, and leather industries as fermentation substrates could provide a low-cost option for the production of microbial enzymes and a variety of other metabolites [62, 92]. Therefore, the use of low-cost waste materials such as keratinous waste such as chicken feathers and animal hairs as fermentation substrate has dual purposes: reducing the fermentation cost for the cultivation of keratinophilic microorganisms (for formulating keratin-based culture media) as well as minimizing the waste load of the environments.

**Figure 7:**
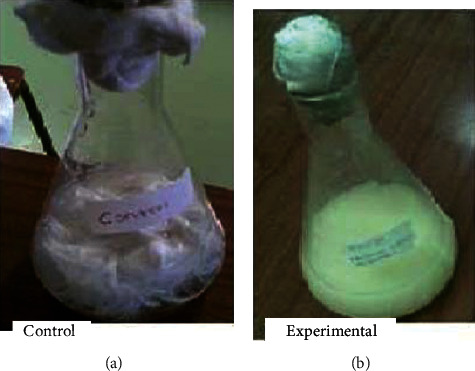
Sheep hair keratinolysis efficiency of *Vibrio* sp. strain R11 isolated from Lake Arenguade, one of the Ethiopian soda lakes, at room temperature: (a) control (flask *Vibrio* sp. strain R11) and (b) completely degraded sheep hair afterward 144 h of fermentation with R11 [62].

Despite many reports revealed, very few microbes have the potential to degrade keratin materials and utilize them as a source of nitrogen and carbon, a representative study [62] showed that keratinous waste (sheep hair) generated from the leather industry particularly from the dehairing stage is used as the sole source of carbon and nitrogen for media component (1% w/v intact hair), instead of using casein, to cultivate keratinophilic microorganism designated as *Vibrio* sp. strain R11 bacteria. Its highest keratinolysis activity was observed when cell growth was at its peak, whereas complete keratin degradation was observed at the end of the stationary phase of the organism. This organism has the capability to degrade intact hair completely into keratin hydrolysate at room temperature within 144 h (Figure 7). This implies that no pretreatment or powdering of the hair was required for the cultivation of *Vibrio* sp. R11. This makes an advantage to reduce growth substrate processing costs. The ability of this organism to grow and produce the appreciable level of keratinase, using hair as a substrate, could be a potential candidate for degradation and utilization of keratinous wastes especially for the development of keratin hydrolysate for different applications. Similar studies have also been carried out by Saber et al. [2] and Lv et al. [93, 94] using animal hair as a source of nitrogen and carbon to cultivate keratinophilic microorganisms. Even if a scarce number of studies have been reported on organisms having the capability to degrade and grow on horn and hoof keratin wastes, some exemplary studies including Balaji et al. [95] proved that a significant amount of keratinolytic enzyme can be produced by *Bacillus subtilis* (MTCC9102) isolate under optimized conditions in solid-state fermentation using horn meal as a substrate.

All the above studies verified the potential use of keratinous wastes as a cheap substrate for the production of keratinase and as a source of soluble hydrolysate that may have potential application in different sectors.

A hair degrading bacterial strain designated as R11 was isolated from around the shore of Lake Arenguade, among the few soda lakes in Ethiopia that contains mud associated with dumped flamingo feathers in decomposition. The proteolytic activity of culture filtrate (crude enzyme) was evaluated using casein as substrate. Phenotypic and genetic analysis results reveal that this bacterium belonged to *Vibrio* sp.

### 5.2. Animal Feed and Food Supplements

Food requirements have risen dramatically in recent years. As a result of this situation, the demand for protein sources for animal feed and dietary supplements has increased. Because keratin hydrolysates from agro-industrial byproducts are mostly consumed by the animal feed industry, keratinous waste recycling is a hot topic in animal nutrition [57, 96, 97]. Yet today keratin wastes are utilized on a limited basis as a dietary protein supplement for animal feedstuffs. Because prior to being used these materials should be hydrolyzed properly without affecting the nutritional composition of the keratin biomass and to make it more digestible. But now, biodegradation of keratin wastes by keratinophillic microorganisms becomes a good opportunity and exemplifies an alternative method to improve the nutritional value of wasted keratin materials by converting the wastes into smaller molecular entities (peptides and amino acids) to be used as feed and food supplements [12]. Similarly, studies [10, 37, 39] found that adding a keratin hydrolysate supplement produced by keratinase or/and keratinolytic microorganisms to animal feedstuff can improve the nutritional value of the feed.

Keratin materials particularly feather, 90% keratin, is a well-known alternative and inexpensive source of protein that can be used in the preparation of animal feedstuff in the form of feather meal [12, 98]. Therefore, to be used as an ingredient of feed keratin materials should be treated with microorganisms or/and their keratinolytic enzymes because biologically treated keratin materials have a much higher digestibility (98%) than the untreated ones (2%). This indicates that keratin materials such as feather hydrolysates can be the potential candidate to be used as a supplementary source of protein in animal feed formulations [66]. For instance, *Brevibacillus* sp. strain AS-S10-II produced an alkaline keratinase that converted feather keratin to essential amino acids such as phenylalanine, valine, lysine, threonine, isoleucine, and methionine [99]. Feather hydrolysate resulted from *Vibrio* sp. strain kr-2 [34] and *Streptomyces* sp. [100] keratinolysis were also reported to improve the nutritional value of feather meals. Likewise, wool protein hydrolysate from *B. pumilus* A1 is also reported as a candidate for animal feed formulation because it has very high digestibility (97%) when compared to that of untreated wool (3%) [101]. Since keratin is naturally poor in some essential amino acids such as methionine and phenylalanine, it has been suggested that using keratinophilic microbial fermentation cultures to enrich the hydrolysate by adding microbial proteins and biomass [10, 34].

Williams et al. [102] reported that keratin hydrolysates obtained from the bacterial fermentation process can have comparable nutritional properties to soybean meal and are a good source of metabolizable protein. As a result, preparing of a meal containing protein obtained from microbially digested keratinous wastes could be one of the cost-effective options to prepare one of the cheapest diet elements. As a result, the ability of keratinase produced from *Vibrio* sp. strain R11 to convert keratin waste to soluble proteins has enormous promise for improving animal feedstuffs. Apart from environmental concerns, the use of keratinolytic protease enzymes in the preparation of soluble peptides and proteins has become increasingly popular. According to reports [62], 3.17 g/L of soluble protein was produced from 10 gm of sheep hair without taking into account the unmeasurable hair protein hydrolysate used as a food for bacterial growth. This means 31.73% of the protein was retrieved. A similar investigation was carried out, yielding 1.44 g/L of soluble protein from disintegrated feathers [103].

### 5.3. Enzyme Production/Keratinase

Although microbes including bacteria, fungi, archaea, and actinomycetes have the ability to produce industrially important enzymes, the cost of their enzyme production is a crucial factor at its industrial scale. In the enzyme industry, growth media is estimated to account for 30% to 40% of the total cost of manufacturing [104].

Keratinous wastes including feather and hair released from poultry, leather, and barbershops can serve as an appropriate growth substrate for microbes and ultimately for the production of keratinolytic enzymes since they are inexpensive and readily available. As a result, the feasibility of producing enzymes on cheap fermentable substrates must be investigated [104].

Keratinase is a protease that can digest insoluble keratin, which is present in keratinous materials such as animal hair, feathers, horns, and hooves more effectively than other proteolytic enzymes [12]. This provides keratinase enzymes, which are industrially significant enzymes that offer bioconversion of keratin wastes. Meanwhile, the application of keratinase at an industrial scale is still in their point of start as compared to other industrial enzymes. Besides this, their market demand is becoming highest currently. With this regard, researches are currently being done [12, 60, 62, 105] to investigate these enzymes from different microbes and utilize them in the processes involving keratin waste hydrolysis.

#### 5.3.1. Keratinolytic Enzyme Production Condition

With the exception of a few thermo-tolerant fungi [106] and bacteria [107] that needs static fermentation conditions, most keratinophilic microorganisms can grow and produce keratinase under submerged shaking conditions. This implies that submerged shaking fermentation is a common method of producing keratinase. However, solid-state fermentation has recently become popular for the production of keratinolytic enzymes [108].

Keratinase is mostly produced in basal media containing mineral salts associated with commercially processed keratin substrate. This indicates that keratin degrading microbes can use processed keratin substrate as their sole carbon and nitrogen source [68, 93]. However, as some investigations have shown, the inclusion of a keratinous substrate is not always necessary for the production of keratinolytic enzymes. The enzyme can be produced by using intact keratinous materials such as feathers and animal hair [62]. Keratinolytic enzymes can be also produced by using other nonkeratinous substances that have an inducing property of keratinolytic enzyme production for the cells [10].

Keratinolytic protease production can be enhanced by supplementation of the media with keratin in addition to other different carbon and/or nitrogen sources. For example, the addition of bagasse [108], molasses [109], and glucose [62, 110] and additional nitrogen sources such as yeast extract, tryptone, urea, peptone, sodium nitrate, and ammonium chloride is reported to increase keratinase production [110].

Keratinase synthesis can be boosted by adding keratin to the media along with other carbon and nitrogen sources. For example, the addition of glucose [62, 110], molasses [109], and bagasse [108], as well as additional nitrogen sources such as peptone, yeast extract, urea, tryptone, ammonium chloride, and sodium nitrate, has been shown to increase keratinolytic enzyme production [110].

As Seid and Gessesse [62] investigated, *Vibrio* sp. strain 11 has the capability to degrade hair and produce keratinase. Hair degradation was carried out in 500 ml Erlenmeyer flasks containing the hair (1% w/v), and basal salt medium components were (g/l): peptone (5), K_2_HPO_4_(1), CaCl_2_ (0.13), yeast extract (1), MgSO_4_.7H_2_O (0.2), and 1% Na_2_CO_3_. A similar study [111] was also conducted using *Paenibacillus woosongensis* TKB2, a feather-degrading, keratinase-producing bacterium. As compared to the media formulated by Seid and Gessesse [62], the media composition hair is replaced with feather, and basal salt medium is slightly modified.

To make keratinolytic enzyme in broth culture media [62], a loop of *Vibrio* sp. seed culture (24 h grown cells) was used. Strain R11 was transferred to the hair basal salt medium (pH = 10) and cultivated for 8 days (192 h) at room temperature using an orbital shaker with 120 rpm. Hair degradation was started at 84 h of incubation (exponential phase) and after the 6^th^ day (144 h) of incubation, complete hair degradation was observed, which was considered late exponential phase. Maximum enzyme production (11.6 U·ml^−1^) was observed at the stationary phase of the strain (8 days). After centrifugation was performed at 10,000 rpm for 5 min, the clear solution (supernatant) was taken as a crude enzyme and used further enzyme characterization and total soluble protein determination. The crude enzymes extracted on the 8^th^ day (the optimal period for keratinase production) and the end of the fermentation period (252 h) were used for further enzyme characterization and total protein determination, respectively.

According to Gupta and Ramnani [37] and Sangali and Brandelli [74], the highest extracellular keratinase enzyme production was reported during the stationary or late exponential phase of microbial development, which was aligned with the investigations of Noval and Nickerson [58]. The production of extracellular keratinolytic enzyme by *Vibrio* sp. strain R11 followed a similar pattern [62]. After 8 days (192 h) of incubation, the highest level of keratinolytic enzyme production was detected. At the late stationary phase, *Vibrio* sp. strain R11 showed a decrease in enzyme production (data not shown), which is consistent with earlier observations [37, 112]. This could be attributed to microbial growth reduction or an increase in cell death linked to the depletion of available nutrients required for R11 cell growth. As an end, no more enzyme was produced, and those that had been produced previously may have gone through enzymatic autolysis and been affected by other end products in the culture media [113]. According to Seid and Gessesse [62], when incubation was prolonged after the cell population reached its stationary phase, the viable cell population of *Vibrio* sp. strain R11 decreased, which could be another cause in the drop of enzyme production during this growth phase. R11 enzyme production remained relatively stable until 252 h, after which it began to decline. As a result, determining the fermentation period for optimal enzyme production requires an understanding of cell development linked with maximum enzyme production. Understanding this issue is also beneficial for using the enzyme in a variety of biotechnological applications.2

#### 5.3.2. General Biochemical Characteristics of Keratinolytic Enzymes

One of the major important factors that must be considered for the production of the industrial enzyme at a large scale is the location of the enzyme with reference to the cell body. If the enzyme is extracellular and secreted into the culture medium, it is preferred for commercial enzyme production because cell disruption is not mandatory, and therefore, the expenses of enzyme preparation are reduced, and it is easy to recover the target enzymes. On contrary, if the enzyme is intracellular and not secreted to the culture medium, there is a need for lysis disruption of the cell, and it is difficult to isolate and purify the enzyme.

Except for few microbial keratinolytic enzymes that are intracellular [114], almost all keratinolytic enzymes are mostly extracellular or secreted into the culture medium [8, 10, 37, 62]. Therefore, these enzymes (keratinases) are preferred for industrial-scale production. As Al-Musallam et al. [115] and Korniłłowicz-Kowalska [116] noted that fungal species particularly *Arthroderma curreyi*, *Arthroderma quadrifidum*, *Chrysosporium pruinosum*, and *Coprinopsis* sp. can produce both extracellular and intracellular keratinase. *Trichophyton gallinae* is an exceptional fungal species that can produce intracellular keratinase [117].

Microbial keratinase is characterized by a great variety in its biophysical and biochemical properties. Because of keratinase-producing microorganisms are isolated from the most distinct soil habitats, including aerobic and anaerobic environments [118]. Due to their source of environmental variability, their optimum pH and temperature also varied. From an industrial point of view, it is fascinating that an enzyme having stable activity in a broad range of pH and temperature is preferable for different operations of industrial processes. Numerous studies including Friedrich and Antranikian and [85] Cheng et al. [109] showed that keratinolytic enzymes have no fixed optimum pH and temperature value. However, most of the researchers agreed that these enzymes work actively from neutral to alkaline conditions and from mesophilic to thermophilic conditions. This indicates that keratinase is catalytically active in a broad range of pH and temperature values. Enzymes showing such kinds of properties particularly keratinases can avoid the need for pH and the temperature control system in the industrial process, and they could be the best choice for different industrial applications.

Almost all keratinase enzymes work efficiently in acidic to alkaline conditions (pH 4.5–11) [10, 39]. Keratinase enzyme produced from *Vibrio* sp. strain R11 [62] also works best at an alkaline condition, pH 11. However, even at extremely alkaline pH, some of them are most active outside of this range such as keratinases produced from alkalophilic bacteria *B. halodurans* AH-101 [119] and *B. circulans* [120] and alkalophilic actinomyces such as *Streptomyces* AB1 [121] and *Nocardiopsis* sp. strain TOA-1 [122] have been found to produce keratinases that perform best in a highly alkaline environment (pH > 11.5).

The optimum temperature of several microbial keratinases is along the thermophilic range of 45–65°C [9, 29, 92, 113, 123, 124]. Keratinase enzymes produced by *Streptomyces* sp. SK1-02, *Scopulariopsis brevicaulis*, *S. pactum* DSM 40530, *B. licheniformis*, and *K. rosea* have similar optimal temperatures between 40 and 50°C [73, 80, 92, 125]. Because of the organism's source and origin, the optimal temperature for these enzymes might range from 30 to 80°C.

Keratinophilic organisms such as *B. circulans* [120], *Thermoactinymces candidus* [82], *Actinomadura keratinilytica* Cpt29 [126], *Fervidobacterium pennavorans* [85], *Thermoanaerobacter keratinoplilus* [127], and *F. islandicum* [128] produce keratinolytic enzymes that work optimally at 70°C or above. Keratinase from *Chrysosporium keratinophilum* [129] exhibited high-temperature optima of 90°C. A unique keratinolytic enzyme produced by *F. islandicum* AW1, which was isolated from a geothermal hot spring, showed exceptionally high-temperature optima at 100°C [107].

Pathogenic organisms such as *Serratia maltophilia* [130], *Serratia marcescens* P3 [131], and *Trichophyton* sp. produce mesophilic keratinases with a lower optimum temperature range (20–45°C) [132]. This is most likely revealing of the place where they inhabit.

Although the molecular mass of various keratinolytic enzymes has been established, their weight falls between 18 and 240 kDa. However, the majority of these enzymes have a molecular weight of less than 50 kDa. Keratinase with metalloprotease properties or those produced by thermo-tolerant organisms are frequently associated with higher molecular mass [10].

Almost all microbial keratinases are serine, metallo, or serine-metallo [39] with the exception of yeast keratinolytic enzyme, which is categorized as an aspartic protease [133]. Lin et al. [69] also reported that most microbial keratinases belong to the serine protease family. Many researches have been also revealed that most *Vibrio* proteases produced by *Vibrio* sp. are serine proteases [62, 134].

The existence of divalent metal ions such as Mg^2+^, Ca^2+^, and Mn^2+^ often excite keratinase activity and increases thermal stabilization of the enzyme. On the other hand, heavy and transition metals such as Hg^2+^, Cu^2+^, Ag^2+^, and Pb2+ generally cause the inactivation of keratinase [10, 107, 135].

Unlike other protease enzymes that become inactive in the presence of bleach-based detergent (NaBO_3_), nonionic surfactant (Tween 80) and organic solvent (methanol) keratinolytic enzymes could have a chance to resist all the above-mentioned chemicals. These chemicals may also activate or stabilize these enzymes [62]. As a result, there will be no denaturation or unfolding of these enzymes. This ability could be attributed to the existence of disulfide bonds in these enzymes, which are required for their activity. This means that the intact disulfide bridge in the polypeptides of the keratinolytic enzymes did not undergo any degradation. The findings suggest that unbroken disulfide bonds are necessary for keratinolytic enzymes to retain the molecular folding required for full activity. Another study verified the role of disulfide bonds in protein stability in the presence of solvents [136].

Proteases with methionine (Met) residues at the catalytic site are easily inactivated by chemical oxidants such as sodium perborate, according to Siezen et al. [137]. As a consequence of Seid and Gessesse's [62] findings, they concluded that the enzyme produced by *Vibrio* sp. R11 has no or few methionine residues in its active site since it has a high capacity to tolerate the bleaching agent's inhibitory effect up to a 15 mM concentration.

#### 5.3.3. Industrial Applications of Keratinolytic Enzymes

Keratinases have recently gained biotechnological impetus (Figure 1) because of their ability to act on hard-to-degrade keratinous wastes such as hair, feather, nail, horn, and hoof and thus becoming a part of solid waste management as recycling of these wastes is tough [118]. The enzymes are also important in processes linked with the biodegradation of keratin waste into animal feed [12] and fertilizers [74, 138]. Other encouraging applications have been associated with keratinase enzymes, including enzymatic dehairing for leather [62], cosmetic preparation [3], detergent formulations [139], and development of biodegradable films or biopolymers [135] from keratin fibers. The use of keratinolytic enzymes to produce bioenergy [10, 140] and enhance drug delivery [141] in some tissues and hydrolysis of prion proteins [142] get up as new outstanding applications for these enzymes.

#### 5.3.4. Leather Industry

**Figure 8:**
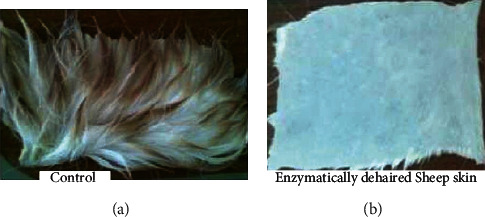
Efficiency of keratinase (58 U/ml), produced by *Vibrio* sp. strain R11, for dehairing activity without any addition of Na_2_S and lime in the reaction mixture at 37°C for 12 h incubation: (a) control (sheepskin treated with denatured or heat-inactivated keratinolytic enzyme at 100°C for 1 h, with buffer alone) and (b) enzyme-treated sheepskin incubated with nondenatured enzyme (normal) [62].

**Figure 9:**
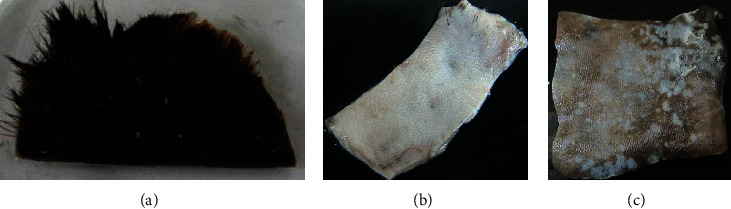
Goatskin dehairing efficiency of keratinase produced by *P. stutzeri* strain K4: (a) skin immersed in distilled water (control), (b) skin immersed in culture filtrate or crude keratinolytic enzyme and after 20 h of incubation (experimental), and (c) goatskin dehaired with chemical (Na_2_S) as a positive control [147].

**Figure 10:**
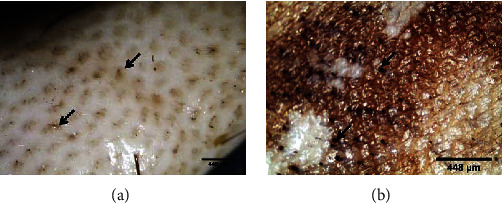
Dehaired goatskin examination result with a stereo microscope under 10x magnification power: (a) dehaired hide after strain k4 culture filtrate (crude keratinase) was applied and (b) dehaired hide after the chemical is applied [147].

**Figure 11:**
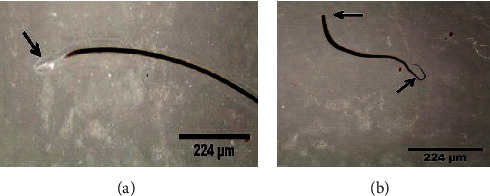
Dehaired goatskin examination result with stereo microscope under 15x magnification: (a) physical status of the hair after the dehairing process using crude keratinolytic enzyme obtained from strain k4 and (b) physical status of the hair after dehairing process by the chemical method [147].

**Figure 12:**
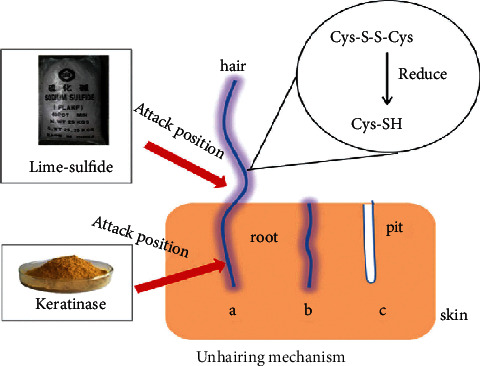
Examination of lime sulfide and keratinase dehairing approaches. Despite they share comparable dehairing mechanism and breakdown of disulfide bonds in the polypeptides, the chemical method attacks the hair shaft outside the skin, and remaining hairs are available on the skin surface, while keratinase attacks the hair root to produce shaft-free skins: Note: a = whole intact hair on the skin without treatment, b = dehairing with chemicals and parts of hair shaft was still remaining on the surface of the hide, and c = dehairing with keratinolytic enzyme and skins are free from any hair shaft on the surface of the hide [148].

Hairs from animal hides are traditionally removed with harsh chemicals such as sodium sulfide and lime. The use of these chemicals in the dehairing process pollutes the environment and poses health risks to leather workers. However, because of their economic and environmental benefits, dehairing by enzyme particularly proteases is currently being developed as an alternative to chemical dehairing and can avoid the use of lime and sulfide in the process [143]. When sulfide is used in the dehairing process, the effluent has an excess chemical oxygen demand (COD) of around 60 g/L. Thus, enzyme-based dehairing techniques minimize or even eliminate the usage of sulfide and lime, resulting in a significant environmental advantage [143–145]. Alkaline proteases are being used more frequently for dehairing, according to Rao et al. [60] and Tanksale [146]. The use of keratinolytic enzyme for leather industry application is not common yet. But some investigations have been conducted for the dehairing purpose including Seid and Gessesse [62], and they found full dehairing activity after 12 h incubation at 37°C using 58 U·ml^−1^ keratinase enzyme, produced by *Vibrio* sp. strain R11 (Figure 8). Interestingly, the experiment was conducted without the addition of lime and sodium sulfide in the reaction mixture to achieve complete dehairing. This was the first report of a novel strain of *Vibrio* sp. strain R11 that shows exceptional dehairing activity and keeps the sheep hide and hairs without any deterioration. Interestingly, the result shows that purification of keratinolytic enzyme prior to dehairing was not mandatory. This is a unique advantage to avoid the cost of enzyme purification. Related work conducted by [147] showed that feather waste can be a source of carbon and nitrogen for the cultivation of *Pseudomonas stutzeri* strain K4 and for the production of keratinolytic enzymes. Crude keratinolytic enzyme exhibited admirable dehairing of goatskin after 20 h of incubation, and intact hairs were obtained without any physical damage. In both studies, the result showed that (Figures 8–12) on dehaired skins (sheep- and goatskins), no hair remains, and the hairs are pulled out rather than cut off from the surface of the skin. This is one criterion in good leather dehairing activity. The results showed that the quality of the hide dehaired with keratinase enzyme is more preferable to that of chemical dehaired hides. The skins dehaired with enzymes are more smooth and clean.

Similarly, Arunachalam and Saritha [149] also observed that full dehairing activity can be achieved by keratinase produced by *B. subtilis* within a 7–9 h incubation interval in the absence of sodium sulfide. This implies that keratinolytic enzymes could be a promising type of biocatalysts to replace Na_2_S in the dehairing process of the leather industry. As a result, using these enzymes is a good approach to decrease the amount of pollution caused by the tannery process when it comes to environmental concerns.

#### 5.3.5. Detergent Industry

It is interesting to note that keratinase-containing detergents can get rid of blood, paint, and color stains from clothes. According to Manivasagan et al. [139], keratinase generated by *Actinobacterium actinoalloteichus* MA-32 has been regarded as a possible candidate for usage in detergent formulation. Similarly, keratinase produced from *B. subtilis* strain RM-01 [150] and from *Vibrio* sp. strain R11 [62] is considered a promising candidate to formulate detergents. However, keratinase produced by *Vibrio* sp. strain R11 is excellent with its stain removal efficiency as compared to the above keratinases. This is because it can remove stain efficiently without the addition of commercial detergent.

**Figure 13:**
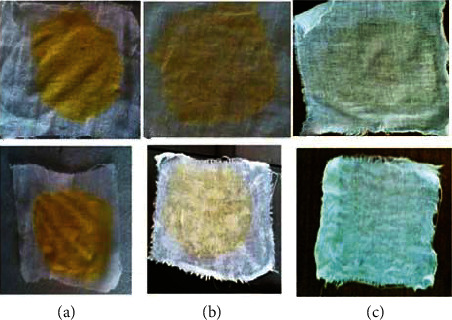
Stain (egg yolk) removal efficiency of commercial detergent (upper pictures) and crude keratinase produced by *Vibrio* sp. strain R11 (lower pictures): (a) nontreated stained cotton fabric, (b) stained cotton fabric treated with 11.6 Uml^−1^ enzyme or 7 mg/ml commercial detergent, 30 min incubation at 37°C [62].

**Figure 14:**
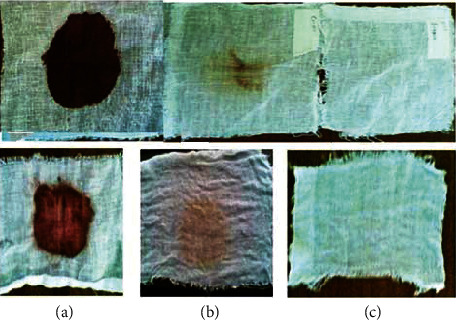
Stain (blood) removal efficiency of commercial detergent (upper pictures) and keratinase produced by *Vibrio* sp. strain R11 (lower pictures): (a) stained cotton fabric before reaction mix, (b) stained cotton fabric treated with glycine/NaOH buffer alone (control), and (c) stained cotton fabric treated with 11.6 Uml^–1^ enzyme or 7 mg/ml commercial detergent, 30 min incubation at 37°C [62].

A cloth made from cotton stained with animal blood and egg yolk was tested without any detergent additives to see if the keratinolytic enzyme produced by *Vibrio* sp. strain R11 could be used for washing (Figures 13 and 14). At room temperature, the enzyme with 5.8 U/ml took 2 h to completely remove both the egg yolk and the bloodstain; however, at 37°C, the stains were removed in 1 h (pictures are not shown). At room temperature and 37°C, complete stain removal took 1 h and 30 min, respectively, when the amount of enzyme was increased to 11.6 U·ml^−1^ (Figures 13 and 14). The enzyme produced by *Vibrio* sp. strain R11 was shown comparable pH stability to commercially important detergent enzymes as Subtilisin Carlsberg and Subtilisin Novo or BPNggs [60].

The enzyme that can be used in the detergent formulation must be catalytically active and stable at high pH (7–11) and temperatures (40–50°C) [112]. Kumar and Takagi [151] also reported that enzymes must be stable and compatible with all widely used commercial detergent ingredients, including oxidizing agents, surfactants, bleaches, and other additives. The enzyme must also be compatible and stable with all commonly used detergent compounds such as surfactants, bleaches, oxidizing agents, and other additives [151]. With this regard, Seid and Gessesse [62] investigated that the keratinase enzyme produced by *Vibrio* sp. strain R11 is efficiently working and very stable in the presence of well-known oxygen bleaching agents (sodium perborate), oxidizing agents (hydrogen peroxide), and nonionic and ionic detergents (SDS). Furthermore, at temperatures below 55°C, the enzyme produced by *Vibrio* sp. strain R11 does not require Ca^2+^ to maintain its activity and stability. This reveals that the enzyme does not require a cofactor such as Ca^2+^ ion even if the temperature is raised during washing activity. All these properties of this enzyme could offer the enzyme has remarkable potential for detergent application.

#### 5.3.6. Silver Recovery Purpose

Silver is an expensive and elegant metal that is utilized in huge amounts for a variety of purposes, particularly in the photographic industry. With an increasing demand for silver in the world, recent attention is focused on used X-ray or photographic films as one of the sources because waste (used) X-ray or photographic films are easily available and cheap resources and are considered the best source for silver recovery with compared to other sources. Gelatin is a product of collagen, an insoluble protein that is not digested by common proteases. Keratinase is a type of protease that can break down gelatin in X-ray film and is thus employed in the silver recovery process [152].

Various methods of silver recovery from used X-ray/photographic films have been practiced. One of these is burning of the films directly. This method generates undesirable foul smells and causes environmental pollution, and base film made of polyester cannot be recovered. Stripping the gelatin-silver layer by chemical methods using various chemicals and reagents causes environmental hazards. Moreover, these methods are time-consuming and expensive and also pose industrial safety problems. Therefore, the methods applied to recover silver from X-ray/photographic waste should be cost-effective and minimize the impact on the environment. Recovery of silver X-ray/photographic waste using enzyme technology can be an alternative option [152, 153]. Therefore, enzymatic hydrolysis of gelatin is an alternative way to extract silver and also to recycle the polyester film base.

**Figure 15:**
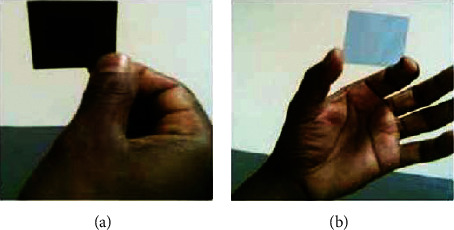
Gelatin removal efficiency of keratinase produced by *Vibrio* sp. strain R11: (a) before and (b) after 3 minutes of incubation at 55°C with 11.6 U/ml enzyme concentrations [62].

The efficiency of keratinase produced by *Vibrio* sp. strain R11 in gelatin layer hydrolysis and silver recovery from used X-ray film was investigated [62]. The hydrolysis of the gelatin layer was maximal at pH 10.5 and 55°C. Complete hydrolysis of gelatin from X-ray film took less than 3 minutes at optimum pH and temperature (Figure 15). The properties of keratinase produced by *Vibrio* sp. strain R11 showed that it has promising potential industrial applications for repeated utilization of the enzyme in both silver recovery and recycling of the polyethylene film base. Similar studies [152, 154] have been conducted to recover silver using protease enzymes, and they have also recommended that enzymes having the capability to hydrolysis gelatin layer from the film are the best alternative method to get well silver from waste/used X-ray and photographic films as well environmentally safe technology.

#### 5.3.7. Keratinnolytic Enzymes in Peptide Synthesis Process

As Gupta [155] explains, synthesis of the peptide by the enzymatic method could have numerous benefits over chemical methods, and therefore, enzymes with organic solvent resistivity are mandatory for the peptide synthesis process because the process needs these solvents in the reaction mixture including methanol. To overcome this problem, keratinolytic enzymes can be a preferable choice because they may work efficiently or withstand high concentrations of methanol. The activity of the keratinolytic enzyme produced by *Vibrio* sp. R11, for example, was increased or activated by adding up to 121% methanol at a concentration of 30%. As a result, this enzyme's methanol compatibility could be used for peptide synthesis and disinfection of microbial contamination at the time of the fermentation process in the presence of a high concentration of methanol. Anaerobic digestion of keratinous waste to generate bioenergy, such as methane and biohydrogen, is another possible application for keratinases [10, 140]. All of the above-reviewed information reveals that keratinolytic enzymes from microbes have attracted a lot of attention in present decades, owing to their wide range of biotechnological applications, including feed, fertilizer, detergent, leather, biomedical science, and other applications.

### 5.4. A Source of Biofertilizer

The use of keratin wastes for the preparation of biofertilizers is becoming one of the potential areas of research today. Because, these materials contain 15–18% nitrogen, 3.2% minerals, 2–5% sulfur, 1.27% fats, and 90% protein, which are essential elements for plant growth [39]. Even though keratinous wastes contain a high amount of nitrogen, they cannot directly be applied as biofertilizer due to the nonavailable form of nitrogen. However, using microbial keratinase, these bioresources can be solubilized into short peptides and amino acids [156].

Fertilizer formulated from keratin wastes such as feather and animal hair hydrolysates allows plants to consume/utilize the nitrogen at their own pace [74, 111, 138]. It is most suitable for plants such as corn, leafy vegetables, and others that require a slow but constant release of fertilizers. Biofertilizers made from keratin wastes can also promote and improve the fertility of cropland fields, soil quality, and soil bioecology and boost the production of plants or products rich in bioactive compounds [3, 157, 158]. Thus, it is clear that such bioresources increase plant growth, mainly in terms of yield and nutritional quality of crops. Using biofertilizers from keratin wastes can be also considered as a substitution for conventional fertilizers, which are expensive for poor farmers as well as the residue of chemical fertilizer may have a chance to pollute aquatic ecosystems [3, 158]. Hence, utilization of keratin wastes as a biofertilizer embodies not only a sustainable keratin waste management method but may also represent an actual approach to tackle environmental pollution.

**Figure 16:**
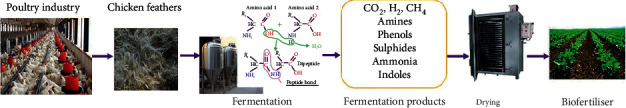
Diagrammatical illustration of biofertilizer preparation from chicken feathers [159].

Since feathers consist of high amounts of nitrogen (>90%) in their structure, it becomes the best alternative biomaterial to be used as biofertilizers (Figure 16). Biodegraded poultry feather hydrolysates may have the potential for the preparation of biofertilizers or soil amendments [25, 98, 160]. Fertilizer produced from feather hydrolysate is characterized as a slow-release, organic, and high-nitrogen fertilizer and is suitable for organic gardens [161]. As [67] reported, fertilizer formed from feather hydrolysate exhibited the same result as that of the commercial fertilizer on the agronomic trait of the two vegetables, Chinese cabbage and carrot.

Microorganisms having the capability to degrade waste biomass of keratin are proposed for use in keratin degradation to prepare keratin-based biofertilizers [158]. Among bacteria, *Bacillus* sp. is considered as the dominant group of bacteria that can degrade keratin wastes by producing a sufficient amount of keratinase, and also actinomycetes play a significant role in the degradation of keratin wastes [68, 125, 161]. Feather waste, for example, can be used as a biofertilizer for ryegrass growth once decomposed with thermophilic actinomycetes strains [162]. Similarly, Seid and Gessesse [62] observed that bacterium *Vibrio* sp. strain R11 has the capability to degrade animal hair completely at room temperature within 144 h, and the bacteria could be the best candidate to formulate biofertilizer from keratinous wastes.

Furthermore, scientific literatures available in Tamreihao et al. [163] including Jeong et al. [130] confirmed that keratinolytic microorganisms and their keratinolysis products can be used to boost agricultural biotechnology. This is because of their biocontrol and plant growth promoter activities of the organisms and their keratin hydrolysis products, For instance, keratinolytic microorganisms and their keratin hydrolysis products can enhance nitrogen-fixing and phosphorus solubilizing bacterial population in the soil and induce the production of indole-3-acetic acid (IAA), which is a plant growth hormone. Therefore, keratinolytic microorganisms and their keratin hydrolysis products, specifically keratin hydrolysates, with antagonistic and plant growth promoter activities could provide a range of economic and environmental benefits over chemical-based fertilizers.

### 5.5. Applications of Keratin Hydrolysate in Tannery Industry

Chromium sulfate is used as a tanning agent in leather processing, and a huge quantity of chromium is released into the effluent, resulting in environmental pollution. Because of its contaminating and hazardous character, the tanning process discharge must be managed and treated to reduce chromium (Cr) exhaustion before it is released for use. As a result, the cost of leather processing in tanneries has increased [164–166]. Furthermore, to increase the required attributes of chrome tanned leathers, retanning is commonly employed to fill the empty nature of the leathers. Different chemicals are utilized as a retanning agent, and this procedure is another step in the tannery process that also pollutes the environment. However, currently, this process operates based on protein-based retanning agents without contributing much load to tannery effluent [167]. Using keratinolytic microbes, hairs and feathers can be bioconverted into keratin hydrolysate, which contains peptides and amino acids, and has been effectively used as a retanning-cum-filling agent in the tannery industry [167–169]. The utilization of biodegraded keratin waste in the retanning process allows for a lubricating effect, which increases the grain smoothness and softness of leathers [167]. As a result, there are two advantages to using keratin hydrolysate in leather manufacturing. It is employed as a filler-cum-retanning agent to replace the leather industry's existing retanning-cum-filling substance, as well as an exhaustive aid in chrome tanning to reduce pollution [165, 166].

For the first time, keratin hydrolysate generated from poultry feathers and animal hair waste from the tannery industry was used to improve Cr exhaustion in tanning and retanning processes at the Central Leather Research Institute (CLRI), Chennai. Keratin hydrolysate (2-3%) can be utilized in chrome tanning (depending on skin weight), and chromium exhaustion in the tanning bath can be as high as 90% [170]. Keratin hydrolysate generated from horn meal by microbial keratinolysis, using *Bacillus subtilis* strain, has also been examined to improve chromium exhaustion in the chrome tanning process and resulted in good insight [95].

### 5.6. Applications of Keratin Hydrolysate in Cosmetic Industry

Because keratin hydrolysates are more compatible with human skin and hair, they have a wide range of uses in the cosmetic industry. Cosmetics that contain keratin-based ingredients have been claimed as a hair and skin treatment [3, 171, 172].

Natural keratin monomeric units can penetrate the skin and hair cuticle and nourish the skin without causing any negative effects. As a result, natural proteins, particularly keratin, are more appropriate for usage or application on human skin and hair. A variety of value-added commercial products such as shampoos, cosmetics, hair conditioners, creams, and biomedical products, can be produced directly from natural keratin hydrolysate since it does not contain any harmful chemicals in its composition [3].

Other natural polymers of keratin include collagen, chitosan, and silk fibroin, which can be used as a vital component of cosmetic blends [173]. Keratin molecules in the stratum corneum and hair cuticle interact with cosmetics to assist the skin to maintain moisture by providing a smoothness and softness sensation. High molecular weight keratin proteins are the most appealing component for skin care treatments because of their hydrophilic and film-forming properties.

### 5.7. Application of Keratin Wastes/Hydrolysates in Textile Industry

Keratin hydrolysate in the form of pure keratin can be used to make nanokeratin-based binders in the textile industry. This material could be used in the pigment printing process without using a cross-linker such as glutaraldehyde [174].

In the textile industry, cotton is commonly dyed with reactive dyes associated with a huge amount of salt and synthetic cationizing agents. Besides increasing the cost of the product and the effluent load, synthetic cationizing agents are nonbiodegradable and cause human health problems since they release toxic chemicals such as formaldehyde and chloroform so that eco-friendly and cost-effective approaches to fabric technique are required [175].

Due to the presence of a large number of reactive amino hydrophilic polar groups (nucleophilic groups) within the molecular structure of cattle hoof and horn keratin, it is possible to synthesize a type of protein derivative agent that can be used as salt-free dyeing auxiliary with reactive dyes in cotton dyeing. Using locally available bioproducts such as animal hoofs and horns as sources of keratin hydrolysate to cationize cotton for salt-free dyeing has dual advantages on the green economy by protecting the environment from the accumulation of the slaughterhouse wastes and by cutting the electrolyte in dyeing, effluent, and wastewater [175].

Chicken feathers are released in large amounts as a byproduct from the poultry industry and are considered valueless materials. However, leftover chicken feather keratin hydrolysate could be employed in the cationization of cloth and subsequent treatment in the textile dyeing process. Feathers waste can be also used in warp yarn sizing and fabric finishing for the production of yarns and fabric [159, 176].

Keratin-based flame retardant material can be synthesized using chicken-feather waste. Chicken-feather keratin is inherently less flammable due to the presence of high doses of nitrogen element within its molecular structures [177]. So using chicken-feather protein as a raw material, which acts as a nitrogen provider, and in combination with other flame retarding monomers and a cross-linking agent would possibly develop a new kind of environmental friendly biological P-N flame retardant to enhance the flame retarding property of the treated cotton fabric. This innovative technique not only could replace the halogenated flame retardants, reduce pollution, and save cost but also could change waste into treasure [178].

To date, feather wastes can be used for infinite applications in different sectors. Few of these produce biodegradable plastics, packaging materials, bioenergy, and materials used in automobile and aeroplane industries. It is also used to create leather composites, biomedical engineering materials, wastewater purification materials, and so on [159]. Because of their drapability, thermal property, warmth, fluff ability, launderability, softness, fire resistance, and durability chicken feather can be used for filling materials in winter clothing and nonwoven fabric production. This implies that the preparation of keratin-based composites and other products with novel characteristics can provide extensive opportunities for bio- and non-biosectors.

### 5.8. Applications of Keratin Hydrolysates for Production of Keratin-Based Biomaterials

Various biopolymers have been used for the preparation of biomaterials, but materials from protein have emerged as potential substitutes for many biomedical and biotechnological applications due to their ability to function as a synthetic extracellular matrix that facilitates cell-to-cell and cell-matrix interactions [105].

One of the natural proteins that does not contain any harmful chemicals and possesses many distinct advantages over conventional biomolecules is keratin. It has unique physico-biochemical characteristics such as biodegradability, withstanding change in temperature during the preparation of biomaterials, mechanical durability, biocompatibility and natural abundance, propensity for self-assembly, and intrinsic cellular recognition. Therefore, due to these properties, keratin is preferred for the preparation of a variety of commercial products including biomaterials. Biomaterials produced from keratin can be used to produce creams, cosmetics, hair conditioners, shampoos, nanokeratin-based binders, and biomedical products [105].

#### 5.8.1. Keratin Biomaterials for Biomedical Applications

**Figure 17:**
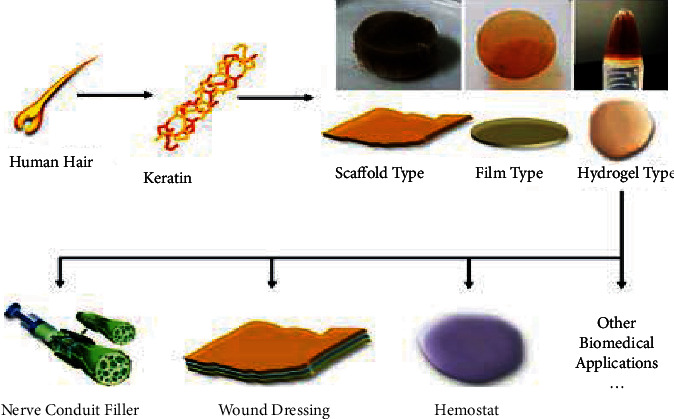
Biomedical applications of human hair keratin-based biomaterials [179].

Keratins have the physical and chemical properties of spontaneous self-assembly and polymerization, allowing for the formation of porous scaffolds, films, and hydrogels, among other biomaterials (Figure 17). Hence, biodegradable polymers such as keratin-based biomaterials can be encouraged as a promising candidate in the field of emerging technologies including biomedical technologies to be used for tissue engineering and renewing medicine, gene therapy, novel drug delivery systems, implantable devices, and nanotechnology [105, 141, 180–182].

Biomaterials prepared from various keratin-containing substances such as wool and human hair have been shown that they are promising for osteoblast differentiation and fibroblast preparation and also for wound-healing purposes. Therefore, biomaterials developed from keratin materials are the promising product to be used as a mitogenic and chemotactic agent for a variety of cell types and to mediate changes in gene expression consistent with the promotion of wound healing [183].

Recently, keratin that is found in human hair has been used for tissue renewal [179] and to enrich human mesenchymal stem cells for medical applications [184]. This makes keratin biomaterial is a potential candidate for cell seeding [181]. Biomaterials or hydrolysates based on keratin can also be utilized to control normal physiological conditions and nerve renewal [105, 183, 185]. Keratin derived from human hair has proven to be an excellent biomaterial with high biocompatibility, no immune response after transplantation, good cellular interaction activity, and biodegradability. The recent development of well-defined and competent procedures for extracting keratin from human hair has resulted in the manufacture of a variety of keratin-based biomaterials (Figure 17), which have been used in successful tissue regeneration approaches [179].

Materials made from wool and human hair in the form of sponge and film already were found in the market for different biomedical applications such as neural tissue engineering and wound dressing applications [182] because these biomaterials have high water uptake capacity of the wound exuding at the wound site and can absorb and maintain moist condition when used as wound dressings and healing to stimulate the growth of cells at the site of damage [181]. The porous keratin sponge biomaterial made from wool and human hair contains a lot of heterogeneous pores and can be used for dermal drug delivery systems particularly for wound dressing and healing (Figure 17).

As Xu et al. [182] and Srinivasan et al. [181] reported, keratin extracted from digested feathers can be used to make protein fibers as well as 2D and 3D scaffolds for tissue engineering. The ability to make self-assemble and polymerize into 3D structures of keratin proteins extracted from keratinous materials has led to their use as scaffolds for tissue engineering [105] and the fabrication of keratin-based composite nanofibers for tissue engineering and regenerative medicine [186]. Due to a large amount of cysteine linkage, films made from chicken feather keratin have good mechanical properties and are used for targeted drug delivery applications [141].

Biofibers in the form of micro- and nanoparticles are successfully regenerated from feather keratin, and they have good biocompatibility and stability [182]. Unlike other nanoparticles, these keratin particles have been found to be water stable and do not require any cross-linking or other chemical modifications, making them acceptable for medical applications. Keratin can be used to make films, hydrogels, fibers, micro- and nanoparticles, and other materials for food, cosmetics, agriculture, composites, textiles, medicine, and other industries [187].

Keratin from horn and hoof is a less explored bioresource for making valuable products including for high-value biomedical applications. However, few studies including [180] approved that keratin extracted from hoof can be one of the alternative biomaterials used for tissue engineering applications because the protein obtained from hoof assures good cell viability.

#### 5.8.2. Development of Wood Adhesives/Glues

As Adhikari et al. [188] reported, wood adhesives can be formulated from partial hydrolysis of proteins. Keratin hydrolyzed peptides show moderate adhesive strength and limited water resistance when used as a wood adhesive. But through chemical cross-linking and blending with phenol-formaldehyde resin or latex-based resins under optimal conditions, peptides-based adhesive formulations may display comparable performance to that of phenol-formaldehyde resins that currently dominate the adhesive market.

#### 5.8.3. Miscellaneous Applications of Keratin Hydrolysates/Keratin Biomaterials

Keratin-derived bioactive peptides have been reported in the literature [189]. Despite their keratin source and the method of preparation, these peptides have a range of activities like inhibition of early stage amyloid aggregation, antimicrobial, anti-inflammatory, antihypertensive, antioxidant, antidiabetic, or anti-aging. For instance, culture supernatants contain bioactive peptides obtained through submerged cultivation of 50 g/L feathers with *Chryseobacterium* sp., strain showing antioxidant properties [190]. Feather hydrolysates were obtained through submerged cultivation of 50 g/L feathers with Chryseobacterium sp. strain kr6. Culture supernatants obtained this organism displaying antioxidant properties [190].

Since feather biomaterials are exceptionally strong and stiff enough, materials made from this keratinous waste might have interesting applications. The blending of keratin (e.g., from chicken feathers) with other biopolymers can be used to develop biocomposites used as reinforcement with their excellent characteristics [186]. Fibers made from feathers can be considered as preferable water filters because they showed better water filtration ability than present common filters, such as those made of activated carbon [156]. Thermoplastic sheets for food packaging and other purposes can also be made from feather keratin [191, 192]. Other keratinous wastes such as horn and hoof hydrolysates can be used to prepare firefighting materials.

### 5.9. Trends of Using Keratin Wastes as Value-Added Products in Ethiopia

Ethiopia is believed to have the largest livestock population in Africa, and a huge amount of keratinous wastes are thrown away to the environment without ample utility and causes pollution. So far, there is no scientific investigation has been conducted with regard to the utilization of keratinous wastes for different modern applications. But Seid and Gessesse [62] try to utilize animal hair as a component of culture media for the cultivation of keratinophilic microorganisms designated as *Vibrio* sp. strain R11 bacteria. This waste is released in huge amount from the leather industry, and no one is considered a usable material for different applications. Therefore, we use this cheap and accessible material used as a source of carbon and nitrogen for the cultivation of bacteria to produce a keratinase enzyme to be used for different applications. Berhe and Wangatia [175] also try to utilize animal hoofs and horns as sources of keratin hydrolysate to cationize cotton for salt-free dyeing in the textile industry.

## 6. Conclusions

This review generally confirmed that keratin waste is an essential biomaterial in the production of a variety of value-added products. The bioconversion of keratinous wastes into commercially useful products would not only protect the environment from pollution but will also boost the economy of various industries. Despite their hard to degrade, keratinous wastes can be efficiently degraded by various keratinophilic microorganisms through the secretion of keratinases. Fungi, actinomycetes, and bacteria are representative microorganisms that have the capability to hydrolysis keratin wastes by secretion of keratinase. Keratin degrader microbes and their keratinase could be used for various applications such as in recycling of keratin waste for environmental protection, leather processing, production of biofertilizer, preparation of cosmetics, formulation of animal feed, detergent formulation, silver recovery, and production of biomaterials used for different applications. Other potential applications of keratinases include the fields of biomedicine, textile, biological control, biodegradable plastic manufacturing, and the generation of green energy, and thus, keratinolytic enzymes can be rightfully called “modern proteases” (Figure 1). Significant amounts of research have been conducted on several keratinous wastes. However, few reports are available on the biodegradation and utilization of keratin from horns and hooves. This implies that keratin from these materials is a less explored source for making valuable products for different applications. The keratinolytic enzyme production media prepared from keratinous waste is considered low-cost and suitable for industrial-scale cultivation of keratinophilic organisms with keratinolytic activity. The inherent physical and chemical properties of keratinous materials have led to the preparation of numerous keratin-based biomaterials that can be used in various applications. Keratin-based biomaterials could give an essential impulse for the engineering of bio-based materials and serve as replacement or improvement of commodity synthetic polymers. The exploitation of the biochemical and physical properties of this abundant and naturally occurring protein has led to various novel uses (Figure 1).
